# Global publication trends and research hotspots of nonalcoholic fatty liver disease: a bibliometric analysis and systematic review

**DOI:** 10.1186/s40064-015-1542-1

**Published:** 2015-12-14

**Authors:** Tong-shuo Zhang, Hua-lei Qin, Tong Wang, Hai-tao Li, Hai Li, Shi-hai Xia, Xiao-hui Xiang

**Affiliations:** Department of Hepatopancreatobiliary and Splenic Medicine, Affiliated Hospital, Logistics University of the Chinese People’s Armed Police Force, 220 Chenglin Road, Hedong District, Tianjin, 300162 China

**Keywords:** Non-alcoholic fatty liver disease, Bibliometrics, Metabolic syndrome, Insulin resistance, Epidemiology, Non-alcoholic steatohepatitis

## Abstract

With the globally increasing prevalence, nonalcoholic fatty liver disease (NAFLD) becomes the predominant cause of chronic liver disease. A global look at the publication trends and the research hotspots of NAFLD are urgently needed to assess the situation of NAFLD research. The global scientific research in the Science Citation Index-Expanded covered articles relevant to NAFLD was retrieved and its bibliometric parameters and research hotspots of NAFLD were systematically evaluated. To sum up, 6356 articles were published in 994 different journals covering 93 SCI subject categories during 1986–2013, in which English was the most predominant language used. Starting from the late 1980s, the publication on NAFLD grew slowly and entered into a highly developing period in the 21st century, especially in the last decade. Besides hepatic steatosis, metabolic syndrome and its combination of symptoms such as obesity, insulin resistance are listed as the top frequent keywords. Bibliometric results suggest that the obviously rapid growth of the articles in recent years appears to be associated with the accelerating incidence of NAFLD and its cofactors such as metabolic syndrome. In addition, epidemiology focusing on comparing different regions and population is attracting ever-growing attention. Meantime, pathology plays an important role in NAFLD research.

## Background

Non-alcoholic fatty liver disease (NAFLD) is a condition with excessive fat accumulation in the liver in individuals who consume little or no alcohol (van der Poorten et al. 2008). In addition to excessive fat, the NAFLD with liver cell injury and inflammation, defined as non-alcoholic steatohepatitis (NASH), which is virtually indistinguishable histologically from alcoholic steatohepatitis (Cohen et al. 2011; LaBrecque et al. 2014). About 30 % of NAFLD progress to NASH, which can lead to fibrosis, cirrhosis, liver failure or even hepatocellular carcinoma (Chalasani et al. 2012). With the globally increasing prevalence, NAFLD becomes the predominant cause of chronic liver disease in many parts of the world (Loomba and Sanyal 2013). With the ever increasing demand for preventing or reversing NAFLD, it is time to have a global look at the history and current situation of NAFLD research.

Bibliometrics, as a main branch of information management field, utilizes quantitative analysis and statistics to describe patterns of publications within a given topic, field, institute, or country (Han and Ho 2011). Bibliometric method has already been applied to medical related topics such as cancer molecular epidemiology (Ugolini et al. 2007), tuberculosis (Ramos et al. 2008), Alzheimer’s disease (Sorensen 2009), acupuncture (Han and Ho 2011), and liver diseases (Qi et al. 2014). As an effective tool for measuring scientific performance as well as making tracks for global trends, Science Citation Index-Expanded (SCI-Expanded) from the Institute for Scientific Information (ISI), is the most frequently used source database for a broad review of scientific accomplishments in a specific field. This study analyzed the languages, subject categories, journals, countries/territories and institutes of articles related to NAFLD in SCI-Expanded from 1986 to 2013 to assess the publication trends and research hotspots in this field using a bibliometric method.

## Method

The data were retrieved from the online version of SCI-Expanded, Web of Science, which indexed 8618 major journals with citation references across 176 scientific disciplines in the year 2014. “*Non alcoholic steatohepatitis”, “non alcoholic fatty liver disease”* and their heteromorphic form as well as their abbreviation limited in liver or hepatology fields were used as keywords to search titles, abstracts, and keywords from 1986 to 2013. All the retrieved results were imported into Excel 2007 for further analysis. The impact factor (IF) of each journal was referred to the JCR in 2013. Contributions of different institutes and countries/territories were estimated by the affiliation of the articles with at least one author. Addresses of the authors determined the collaboration type. All the articles within the past 28 years (1986–2013) were assessed by the following aspects: document type and language of articles; distribution of journals and subject categories; publication productions of article countries/territories and institutes with five indicators including total publications, single-country publications, internationally collaborative publications, first author articles, and corresponding author articles. Furthermore, the top ten frequently cited articles about NAFLD in SCI-Expanded database were also discussed.

Some of the keywords such as “NAFLD”, “NASH” and “fatty liver” were discarded for the reason that they completely overlap with the research content. At the same time, some key words with the same meanings were unified into one since non-standardized format of key words might bring about statistic bias. For instance, “diabetes mellitus” and “diabetes” were pooled in one keyword, so were “hepatic fibrosis” and “liver fibrosis”.

## Results

### The distribution of publication type and language

There were 11,751 publications with 13 document types indexed in the SCI-Expanded from 1986 to 2013, which included 6356 articles. The original article, as the most popular document type, comprised 54.1 % of the total publications followed by meeting abstracts (2859; 24.3 %), reviews (1289; 11 %), letters (485; 4.1 %), editorial materials (440; 3.7 %), proceedings papers (250; 2.1 %). The remainder having less significance were corrections (36), book reviews (20), news items (9), book articles (4), reprints (1), notes (1) and addition correction (1). Only articles were included for further analysis, in which 14 languages were used. English was the most used language (6216; 97.80 %), and other languages frequently used were French (40; 0.63 %), Spanish (39; 0.61 %) and German (31; 0.49 %). Other languages such as Russian, Portuguese, Polish, Turkish only account for 0.47 % of the total articles.

### Increase pattern of the article amount

**Fig. 1:**
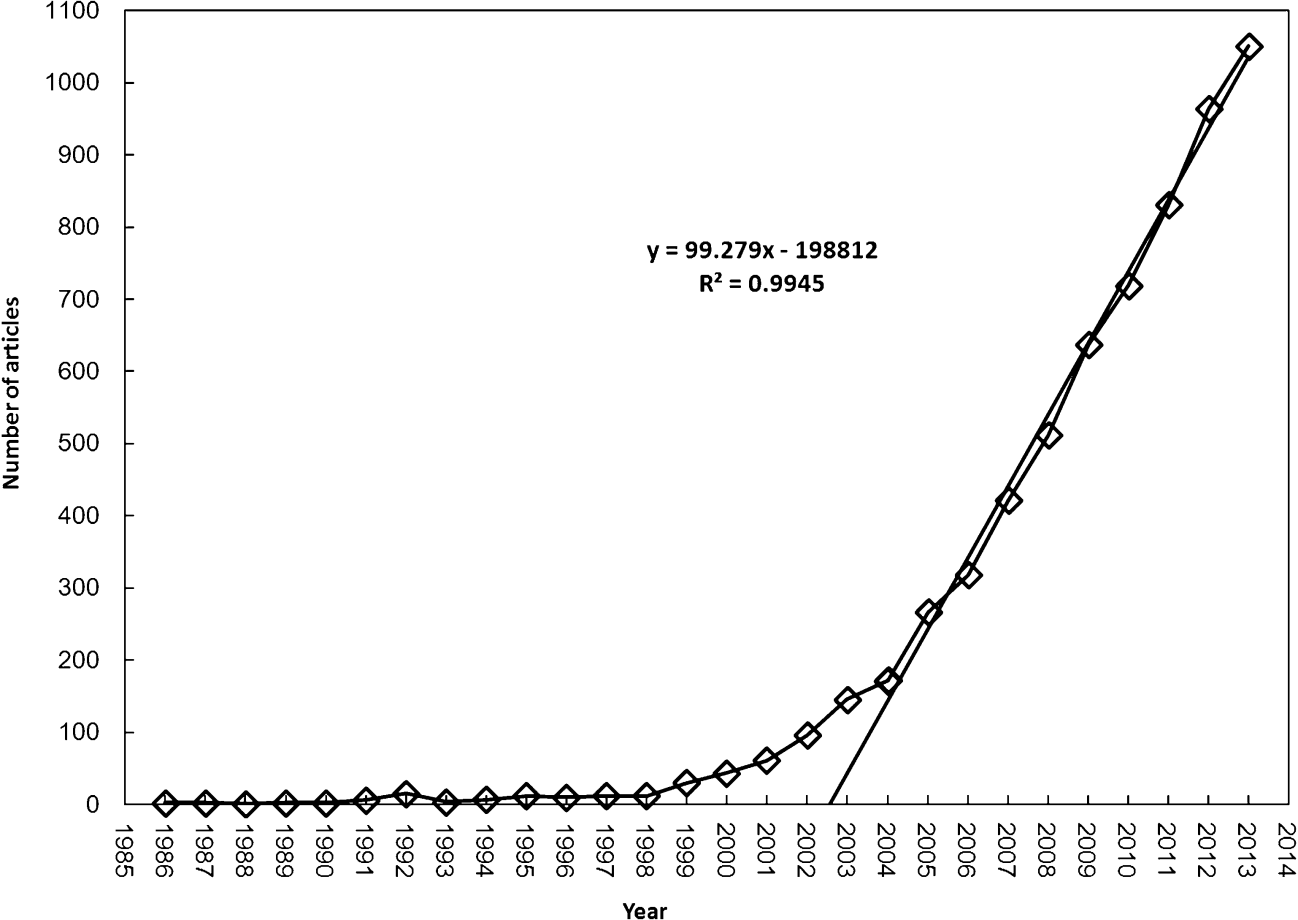
Number of global SCI articles varies with time. Fitting equation during 2004–2013 is: Y = 99.279X − 198812, R^2^ = 0.9945. In the equation, Y is the number of accumulation articles and X is the sequence number of the year. It indicated that research on NAFLD developed rapidly in recent 10 years

After being screened, the meaningful research articles about NAFLD appeared in 1986. The articles in this field kept a very small scale during the period of 1986 to 1998. From 1998 to 2004, the total amount of NAFLD articles experienced a slowly increase. From 2004 to 2013, the total amount of articles in this field entered a period of rapid growth (Fig. 1). According to the growth trend, a power functional equation can be fitted as follows: Y = 99.279X − 198812, R^2^ = 0.9945.

### Subject categories and journals

According to the JCR in 2013, the article output of NAFLD were distributed in 93 SCI subject categories. The top ten main subject categories were Gastroenterology & Hepatology (2655, 41.8 %), Endocrinology & Metabolism (696, 11.0 %), Biochemistry & Molecular Biology (427, 6.7 %), Nutrition & Dietetics (416, 6.5 %), Pharmacology & Pharmacy (352, 5.5 %), Research & Experimental Medicine (329, 5.2 %), General & Internal Medicine (323, 5.1 %), Surgery (277, 4.4 %), Cell Biology (201, 3.2 %), and Pathology (201, 3.2 %).

Totally, 6356 articles were published in 994 journals including professional journals and journals of other disciplines. Among the 994 journals, 428 (43 %) journals only published one article and 166 (17 %) journals published two articles so far. Thirty percent of articles were published in 13 core journals (Table 1). The most productive journal was Hepatology (356), followed by Journal of Hepatology (261), Plos One (167), World Journal of Gastroenterology (163), Journal of Gastroenterology and Hepatology (151), and Liver International (132).

**Table 1:** The 13 most productive journals with the number of articles, IF, SCI categorie of journals, and the position of the journal in its category

Journal	TP (%)	IF	SCI subject category	Position
Hepatology	356 (5.60)	11.190	Gastroenterology & Hepatology	3/75
Journal of Hepatology	261 (4.10)	10.401	Gastroenterology & Hepatology	5/75
Plos One	167 (2.63)	3.534	Multidisciplinary sciences	8/55
World Journal of Gastroenterology	163 (2.56)	2.433	Gastroenterology & Hepatology	36/75
Journal of Gastroenterology and Hepatology	151 (2.38)	3.627	Gastroenterology & Hepatology	19/75
Liver International	132 (2.08)	4.447	Gastroenterology & Hepatology	14/75
Digestive Diseases and Sciences	129 (2.03)	2.55	Gastroenterology & Hepatology	32/75
Gastroenterology	108 (1.70)	13.926	Gastroenterology & Hepatology	1/75
Hepatology Research	99 (1.56)	2.218	Gastroenterology & Hepatology	41/75
American Journal Of Gastroenterology	95 (1.49)	9.213	Gastroenterology & Hepatology	6/75
Obesity Surgery	84 (1.32)	3.739	Surgery	15/204
European Journal of Gastroenterology & Hepatology	76 (1.20)	2.152	Gastroenterology & Hepatology	48/75
American Journal of Physiology-Gastrointestinal and Liver Physiology	76 (1.20)	3.737	Gastroenterology & Hepatology	17/75

*TP* total publications, *IF* impact factor

### Publication trends of countries/territories

**Fig. 2:**
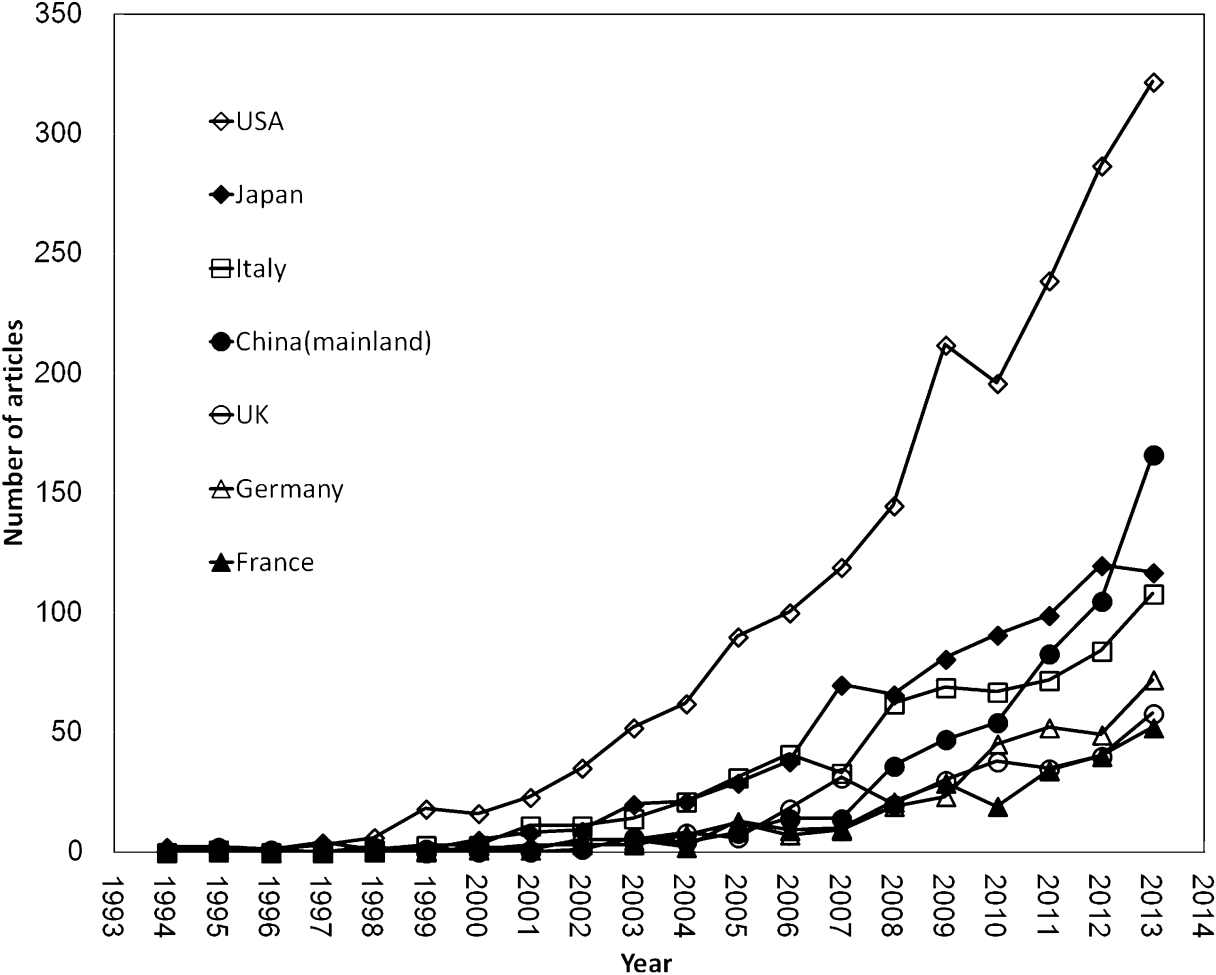
Comparison of the NAFLD publication growth trends of top seven countries

Top 11 countries/territories have published over 200 articles, which were ranked in accordance with the number of total articles and other five indicators (Table 2). The most productive country was the USA (1995, 25.61 %), followed by Japan (802, 10.30 %), Italy (663, 8.51 %), and China (mainland) (549, 7.05 %). The USA had the most numerous partners with 23.43 % of the internationally collaborative articles, followed by Italy with 9.09 %. In addition, the USA ranked the top for its dominant first author articles (26.15 %) and corresponding author articles (26.47 %). The UK (56.17 %) had a relatively high percentage of internationally collaborative articles, while Germany (53.07 %) and Australia (52.91 %) were sequenced behind. The time series analysis among the top seven countries/territories with more than 300 articles is shown in Fig. 2. The number of articles of the USA and China had a sharp increase in recent years. It is worth mentioning that China (mainland) topped Italy in 2010, and then, leapt over Japan in 2012 to be the second place. Moreover, some East Asian countries/territories occupied an important place in the research of NAFLD such as Japan, China (mainland), South Korea and Taiwan.

**Table 2:** Research publication trends of country/territories

Country/territory	TP	TPR (%)	SPR (%)	CPR (%)	FAR (%)	RPR (%)	% C
USA	1995	1 (25.61)	1 (26.69)	1 (23.43)	1 (26.15)	1 (26.47)	29.32
Japan	802	2 (10.30)	2 (12.91)	6 (4.92)	2 (11.59)	2 (11.62)	15.34
Italy	663	3 (8.51)	3 (8.38)	2 (9.09)	3 (8.52)	3 (8.46)	34.24
China (mainland)	549	4 (7.05)	4 (7.48)	5 (6.20)	4 (7.54)	4 (7.51)	28.23
Germany	326	5 (4.16)	9 (2.89)	4 (6.93)	7 (3.61)	7 (3.61)	53.07
UK	324	6 (4.11)	10 (2.68)	3 (7.29)	9 (3.25)	10 (3.12)	56.17
France	313	7 (4.02)	6 (3.95)	8 (4.32)	6 (3.82)	6 (3.85)	34.50
Turkey	253	8 (3.25)	5 (4.46)	23 (0.68)	5 (3.83)	5 (3.83)	6.72
South Korea	245	9 (3.15)	7 (3.50)	12 (2.40)	8 (3.34)	8 (3.34)	24.49
Spain	239	10 (3.07)	8 (3.06)	10 (3.08)	10 (3.20)	9 (3.18)	32.22
Australia	223	11 (2.86)	13 (1.98)	7 (4.72)	11 (2.49)	11 (2.45)	52.91

*TP* the number of total publications, *TPR (%)* the rank and percentage of total publications, *SPR (%)* the rank and percentage of single-country publications, *CPR (%)* internationally collaborative publications, *FAR (%)* first author articles; *RPR (%)* corresponding author publications in total articles, *%C* country collaboration ratio, the percentage of collaborative articles in total articles for each country

### Publication trends of institutes

With 95 publications, University Calif San Diego (USA) ranked the first place, which accounted for 1.51 % of the number of total articles retrieved in NAFLD research. Among the top 10 institutions, 7 of them were derived from USA, publishing 727 articles in total, which accounted for 11.58 % of the number of total articles retrieved. This indicated that the number of articles published presented a centralized trend as in Table 3. Overall, 6356 articles were completed by 4136 institutions, each of which published 1.5 papers on average. Out of the articles, 2326 (36.60 %) articles were completed by single institutions and 3952 (62.18 %) through inter-institutional cooperation. Therefore, cooperation was becoming an inevitable trend in NAFLD research.

**Table 3:** Top 10 most productive institutes of articles during 1986–2013

Institute	R (TP)	R (SP)	R (CP)	R (FA)	R (RP)	% C
University Calif San Diego, USA	1 (95)	13 (16)	1 (79)	1 (56)	1 (50)	83.16
Mayo Clin, USA	2 (80)	1 (26)	10 (54)	2 (53)	2 (49)	67.50
Johns Hopkins University,USA	3 (73)	4 (20)	12 (53)	14 (33)	19 (26)	72.60
University Turin, Italy	4 (72)	122 (4)	2 (68)	60 (16)	47 (17)	94.44
Duke University,USA	5 (71)	21 (13)	4 (58)	4 (42)	3 (42)	81.69
Washington University,USA	6 (70)	13(16)	10 (54)	9 (37)	7 (35)	77.14
University Milan, Italy	7 (69)	31 (11)	4 (58)	6 (38)	12 (30)	84.06
University Sydney, Australia	8 (67)	31 (11)	6 (56)	10 (36)	16 (27)	83.58
University Washington, USA	9 (65)	44 (9)	6 (56)	81 (14)	70 (14)	86.15
University Calif San Francisco, USA	9 (65)	44 (9)	6 (56)	18 (30)	16 (27)	86.15

*TP* the number of total publications, *SP* the number of single institute publications, *CP* inter-institutionally collaborative publications, *FA* first author publications, *RP* corresponding author publications, *R* rank, *%C* the percentage of collaborative articles in total articles for each institute

### The most frequent cited articles

Generally, citation frequency of articles could reflect the foci and the trends of research in a specific area. Analysis of the top 10 most frequently cited articles showed that they were respectively from USA (5 articles), Italy (4 articles) and Australia (1 articles) (Table 4). Four articles were related to epidemiological studies of NAFLD, which were published respectively in the year of 1990 (cited 813 times), 1999 (cited 731 times), 2004 (cited 1029 times), and 2005 (cited 776 times). These articles investigated the similarities and differences in different regions and races, in which the potential role of genetics, the risk factors, and independent predictors of NAFLD were explored. Another three articles were related to the relationship between NAFLD and metabolic syndrome, in which obesity and insulin resistance were repeatedly mentioned. In addition, the remain three articles gave an analysis of NAFLD in the clinical and histological aspect. Two out of three articles explored the histological grading and staging of NAFLD.

**Table 4:** The information of top 10 most frequently cited articles

Name of articles	Journal	First author/institute	Year	Times cited
Design and validation of a histological scoring system for nonalcoholic fatty liver disease	Hepatology	Kleiner, DE/National Academy of Sciences, USA	2005	1575
Nonalcoholic steatohepatitis: a proposal for grading and staging the histological lesions	American Journal of Gastroenterology	Brunt, EM/Saint Louis University, USA	1999	1391
Prevalence of hepatic steatosis in an urban population in the United States: impact of ethnicity	Hepatology	Browning, JD/Donald W. Reynolds Cardiovascular Clinical Research Center, USA	2004	1029
Nonalcoholic fatty liver, steatohepatitis, and the metabolic syndrome	Hepatology	Marchesini, G/Università di Bologna, Bologna, Italy	2003	970
Nonalcoholic fatty liver disease—a feature of the metabolic syndrome	Diabetes	Marchesini, G/Università di Bologna, Bologna, Italy	2001	914
The natural-history of nonalcoholic steatohepatitis—a follow-up-study of 42 patients for up to 21 years	Hepatology	POWELL, EE/University of Queensland and Department of Pathology, Australia	1990	813
The natural history of nonalcoholic fatty liver disease: a population-based cohort study	Gastroenterology	Adams, LA/Mayo Clin, CollMed, USA	2005	776
Independent predictors of liver fibrosis in patients with nonalcoholic steatohepatitis	Hepatology	Angulo, P/Mayo Clinic and Foundation, USA	1999	731
Association of nonalcoholic fatty liver disease with insulin resistance	American Journal of Medicine	Marchesini, G/Università di Bologna, Bologna, Italy	1999	677
Expanding the natural history from cryptogenic cirrhosis to of nonalcoholic steatohepatitis: Hepatocellular carcinoma	Gastroenterology	Bugianesi, E/University of Turin, Italy	2002	624

### The research hotspots of NAFLD

Statistically, 7011 keywords appeared 17,959 times and 5281 keywords appeared only once. Table 5 shows the top 11 keywords with a frequency of at least 100 times. Not all of the articles were included in this analysis because 1978 (31 %) articles were short of keywords. The analysis of the keywords frequency related to NAFLD indicated that metabolic syndrome (MS) and its combination of symptoms (obesity, insulin resistance, oxidative stress, adiponectin, and type 2 diabetes, etc.) were involved in 2029 (11 %) times. Among the frequency of MS related keywords, 579 (2.9 %) times were associated with obesity and 488 (2.4 %) times with insulin resistance (IR). Other topics discussed were histological characteristics and progression of NAFLD (1344, 8 %). Furthermore, a similar result was shown in the analysis of the 10 most frequently cited articles.

**Table 5:** Eleven most frequent key words during 1986–2013 (frequency >100)

Rank	Keyword	Frequency (%)
1	Hepatic steatosis	580 (3.2)
2	Obesity	579 (3.2)
3	Insulin resistance	488 (2.7)
4	Metabolic syndrome	422 (2.3)
5	Liver fibrosis	368 (2.0)
6	Oxidative stress	269 (1.5)
7	Inflammation	128 (1.0)
8	Hepatitis C virus (HCV)	145 (0.8)
9	Adiponectin	138 (0.8)
10	Hepatocellular carcinoma	133 (0.7)
11	Diabetes	133 (0.7)

## Discussion

In this article, bibliometrics was used to quantitatively analyze global publication trends and research hotspots of NAFLD. The publication amount, to a certain extent, can reflect the development level in NAFLD research field.

### Development of NAFLD-related publications

Increased amount of published articles about NAFLD reflects the trend of the disease. NAFLD has been increasing worldwide over recent decades in line with the increased prevalence of obesity, diabetes, and hyperlipemia (Neuschwander-Tetri 2005). This indicates that research on NAFLD would develop more rapidly in the near future because modern sedentary and over-nutrition lifestyle puts a very large population at risk of NAFLD (Farrell et al. 2013).

It is worth mentioning that China (mainland) topped Italy in 2010, and then, leapt over Japan in 2012 to be the second place. Moreover, some East Asian countries/territories such as Japan, China (mainland), South Korea, and Taiwan occupied a more and more important place in the NAFLD research. The rapid increase of publications might be closely related to the ever-growing incidence rate of NAFLD in Asia in recent years, the advance of scientific research, and the growth of economic power that enabled these countries/territories to put more effort in the research and control of NAFLD (Farrell et al. 2013).

Keywords used in bibliometrics could trace the direction and breakthrough of NAFLD research. The frequency analysis of keywords related to NAFLD indicated that metabolic syndrome (MS) and its combination of symptoms (obesity, insulin resistance, oxidative stress, adiponectin, diabetes, etc.) were closely involved in the pathogenesis of this disease. Other topics discussed were histological characteristics and progression of NAFLD in respect that the diagnosis of NAFLD depend on liver biopsy (LaBrecque et al. 2014), which remains the gold standard for the diagnosis of NASH (Fan and Farrell 2009).

In all types of publications, meeting abstracts and reviews account for more than one third of the publications, which are relatively high in proportion. Three reasons might explain the low proportion of articles in all NAFLD publications. Firstly, the still uncertain pathogenesis of NAFLD might hinder the people from researching in this filed (Wu et al. 2011). Secondly, no suitable animal models replicating the full spectrum of the disease in humans might limit the basic research in this field. No uniformed diagnosis guidelines and no established therapy, especially no adequate prospective, double-blind, controlled trials to provide the data necessary to create an evidence-based clinical guideline (LaBrecque et al. 2014), might be the third reason for the high proportion of meeting abstracts and reviews.

### Incidence and prevalence of FAFLD

The obviously rapid growth of the articles in recent years might reflect the accelerating incidence of NAFLD. Contrary to the prevalence of other chronic liver diseases which remained stable or even decreased, the prevalence of NAFLD has doubled during last two decades. NAFLD and NASH, the number one culprit of liver disease in western countries, play a similar important role in the Middle East, Far East, Africa, the Caribbean, and Latin America more recently (LaBrecque et al. 2014). The reported prevalence of NAFLD also varies widely depending on the definition used and the population studied (Williams et al. 2011; Lee et al. 2007; Vernon et al. 2011; Browning et al. 2004; Lazo et al. 2013; Fleischman et al. 2014). The worldwide prevalence of NAFLD has been estimated at 20–30 % (Lopez-Velazquez et al. 2014), and 2–3 % of adults have NASH (Neuschwander-Tetri 2005). In western countries, NAFLD prevalence is up to 90 % in morbidly obese individuals and NASH is rising to 37 % of the morbidly obese (Bedogni et al. 2005). In Asia, the prevalence of NAFLD is reported to be 12–24 % (Fan et al. 2007). In China, the prevalence of NAFLD was about 15 % in adults in Shanghai, Guangzhou and Hong Kong (Fan et al. 2011). In parallel with the epidemic of obesity and metabolic syndrome worldwide, the prevalence of NAFLD in Asian countries has increased rapidly with a trend to younger patients during the last two decades (Fan et al. 2007). NAFLD rapidly becomes the most common form of chronic liver disease in the pediatric population (Lindback et al. 2010).

### Potential pathogenesis of NAFLD

The research hotspots extracted from bibliometrics information of NAFLD reflect the potential pathogenesis of the disease. The analysis of the frequency of keywords related with NAFLD indicated that metabolic syndrome (MS) and its combination of symptoms such as obesity, insulin resistance and diabetes as well as oxidative stress and dyslipoproteinemia were closely involved in the pathogenesis of this disease. Indeed, although pathogenesis of NAFLD remains not fully understood, it is generally attributed to the occurrence of insulin resistance, lipid metabolism dysfunction, oxidative stress, inflammation, and necro-apoptosis, which is consistent with bibliometrics analysis results (Xiao et al. 2013; Vuppalanchi and Chalasani 2009; Rector et al. 2008).

NAFLD/NASH is increasingly regarded as a hepatic manifestation of metabolic syndrome, and the severity of NAFLD seems to increase in parallel with other features of metabolic syndrome (Boppidi and Daram 2008; Liu et al. 2010; Marchesini et al. 2003). Thus, it is evident that pathogenesis focusing on metabolic syndrome has been the prevalent direction of researches. However, not all patients with these conditions have NAFLD/NASH, and not all patients with NAFLD/NASH suffer from one of these conditions (LaBrecque et al. 2014).

### Animal model of NAFLD

No suitable animal models replicating the full spectrum of the disease in humans might hinder the basic research in this field. Animal models that simulate certain features of the human disease have provided insights into possible pathological mechanisms contributing to its development. Animal models used to study the pathogenesis of NAFLD are generally divided into genetically altered, diet induced and combination types (Takahashi et al. 2012; Fan and Qiao 2009; Kanuri and Bergheim 2013). Genetic models of NAFLD include the animals with deficiency in leptin signaling, hepatic lipogenesis, β-oxidation, NF-κB and TNF signaling, and cholesterol signaling etc. (Anstee and Goldin 2006; Kanuri and Bergheim 2013; Nagarajan et al. 2012). Nutritional models of NAFLD include methionine- and choline-deficient model, high fat diet model, atherogenic diet model, fructose model and overnutrition model etc. (Jeong et al. 2005; Spruss et al. 2009; Tipoe et al. 2009). Because of the occurrence and progression of NAFLD/NASH in human being a long period of several decades and its ethical limitations, animal models of NAFLD/NASH give crucial information, not only in elucidating the pathogenesis of NAFLD/NASH but also in examining therapeutic effects of various agents (Takahashi et al. 2012). An ideal model of NAFLD/NASH should correctly reflect both hepatic histopathology and pathophysiology of human NAFLD/NASH, which will boost the research on this disease.

### Treatment progress for NAFLD

Shortage of uniformed diagnosis guidelines and no established therapy might also hinder the research in this field. It is found that mortality increased only in NASH patients but not in patients with common liver steatosis after long-term follow-up of patients with NAFLD. So only the patients with NASH should be considered to receive medical treatment, particularly those who present evidence of fibrosis (Machado and Cortez-Pinto 2014). The primary goal of NAFLD therapy recommended is to prevent the existing comorbidities such as metabolic disorders, cardiovascular or cerebrovascular events, while the reversal of hepatic steatosis is the secondary target for NAFLD treatment (Basaranoglu and Ormeci 2014; Chalasani et al. 2012; Ekstedt et al. 2006). Additionally, prevention and treatment of NASH, control the progression of liver disease, and reducing the occurrence of liver cirrhosis should also be considered (Machado and Cortez-Pinto 2014).

Lifestyle intervention including weight reduction, dietary modification and physical exercise is critical in any attempt to reverse the course of NAFLD/NASH (Chalasani et al. 2012; LaBrecque et al. 2014). Lifestyle modification with diet and exercise is the base of pharmacological treatment (Agrawal and Duseja 2012). For pharmaceutical therapies, a wide range of drugs, including antioxidants, insulin sensitizers, lipid lowering agents, and rennin-angiotensin system blockers, have been applied in clinical trials (Gossard and Lindor 2011; Della et al. 2011; Xiao et al. 2013). In the Pioglitazone or Vitamin E for NASH Study (PIVENS), investigators compared pioglitazone or vitamin E treatments to placebo. This largest placebo-controlled, randomized clinical trial of therapies ever conducted for NASH provides key evidence to support that vitamin E and pioglitazone could help certain patients with NASH (Sanyal et al. 2010). NAFLD or NASH per se is not an indication for bariatric surgery, but there is ample evidence to show that sustained weight loss associated with bariatric surgery can improve and even reverse changes of NAFLD and NASH (Hafeez and Ahmed 2013; LaBrecque et al. 2014). Liver transplantation could be considered for patients with NASH complicated by liver failure, decompensated cirrhosis and hepatocellular carcinoma (Fan et al. 2011). Herbal treatment is likely to offer certain health benefits without obvious adverse effects in NAFLD therapy in the past decades (Xiao et al. 2013). However, detailed mechanistic researches and long term clinical evaluations for Chinese medicine treatment of NAFLD are needed for their future applications (Shi et al. 2012; Xiao et al. 2013).

### Limitations

In order to ensure a high quality bibliometric analysis, we only recruited papers published in SCI-Expanded journals, which has to pay a price that a considerable amount of papers published in non-SCI journals. Especially, the vast amount of herbal treatment literature is published in Chinese. To compensate this pitfall, another study aiming to systemically review the non-SCI papers is needed to make a thorough review on papers related with NAFLD from bibliometric point of view.

## Conclusions

Bibliometric results suggest that the obviously rapid growth of the articles in recent years appears to be associated with the accelerating incidence of NAFLD and its cofactors such as metabolic syndrome. Among the research hotspots of NAFLD, insulin resistance, a common factor in the metabolic syndrome, might play a major role in the pathogenesis research of NAFLD. In addition, epidemiology focusing on comparing different regions and population is attracting ever-growing attention. Meantime, pathology plays an important role in NAFLD research. With the research on NAFLD booming, a more effective way to prevent or control the global prevalence of NAFLD will be expected in the near future.

## Abbreviations

NAFLD: Nonalcoholic fatty liver disease
NASH: Non-alcoholic steatohepatitis

## References

[CR1] Agrawal S, Duseja AK (2012). Non-alcoholic fatty liver disease: east versus west. J Clin Exp Hepatol.

[CR2] Anstee QM, Goldin RD (2006). Mouse models in non-alcoholic fatty liver disease and steatohepatitis research. Int J Exp Pathol.

[CR3] Basaranoglu M, Ormeci N (2014). Nonalcoholic fatty liver disease: diagnosis, pathogenesis, and management. Turk J Gastroenterol.

[CR4] Bedogni G, Miglioli L, Masutti F, Tiribelli C, Marchesini G, Bellentani S (2005). Prevalence of and risk factors for nonalcoholic fatty liver disease: the Dionysos nutrition and liver study. Hepatology.

[CR5] Boppidi H, Daram SR (2008). Nonalcoholic fatty liver disease: hepatic manifestation of obesity and the metabolic syndrome. Postgrad Med.

[CR6] Browning JD, Szczepaniak LS, Dobbins R, Nuremberg P, Horton JD, Cohen JC, Grundy SM, Hobbs HH (2004). Prevalence of hepatic steatosis in an urban population in the United States: impact of ethnicity. Hepatology.

[CR7] Chalasani N, Younossi Z, Lavine JE, Diehl AM, Brunt EM, Cusi K, Charlton M, Sanyal AJ (2012). The diagnosis and management of non-alcoholic fatty liver disease: practice Guideline by the American Association for the Study of Liver Diseases, American College of Gastroenterology, and the American Gastroenterological Association. Hepatology.

[CR8] Cohen JC, Horton JD, Hobbs HH (2011). Human fatty liver disease: old questions and new insights. Science.

[CR9] Della CC, Alisi A, Iorio R, Alterio A, Nobili V (2011). Expert opinion on current therapies for nonalcoholic fatty liver disease. Expert Opin Pharmacother.

[CR10] Ekstedt M, Franzen LE, Mathiesen UL, Thorelius L, Holmqvist M, Bodemar G, Kechagias S (2006). Long-term follow-up of patients with NAFLD and elevated liver enzymes. Hepatology.

[CR11] Fan JG, Farrell GC (2009). Epidemiology of non-alcoholic fatty liver disease in China. J Hepatol.

[CR12] Fan JG, Qiao L (2009). Commonly used animal models of non-alcoholic steatohepatitis. Hepatobiliary Pancreat Dis Int.

[CR13] Fan JG, Saibara T, Chitturi S, Kim BI, Sung JJ, Chutaputti A (2007). What are the risk factors and settings for non-alcoholic fatty liver disease in Asia-Pacific. J Gastroenterol Hepatol.

[CR14] Fan JG, Jia JD, Li YM, Wang BY, Lu LG, Shi JP, Chan LY (2011). Guidelines for the diagnosis and management of nonalcoholic fatty liver disease: update 2010: (published in Chinese on Chinese Journal of Hepatology 2010; 18:163-166). J Dig Dis.

[CR15] Farrell GC, Wong VW, Chitturi S (2013). NAFLD in Asia—as common and important as in the West. Nat Rev Gastroenterol Hepatol.

[CR16] Fleischman MW, Budoff M, Zeb I, Li D, Foster T (2014). NAFLD prevalence differs among hispanic subgroups: the multi-ethnic study of atherosclerosis. World J Gastroenterol.

[CR17] Gossard AA, Lindor KD (2011). Current therapies for nonalcoholic fatty liver disease. Drugs Today (Barc).

[CR18] Hafeez S, Ahmed MH (2013). Bariatric surgery as potential treatment for nonalcoholic fatty liver disease: a future treatment by choice or by chance. J Obes.

[CR19] Han JS, Ho YS (2011). Global trends and performances of acupuncture research. Neurosci Biobehav Rev.

[CR20] Jeong WI, Jeong DH, Do SH, Kim YK, Park HY, Kwon OD, Kim TH, Jeong KS (2005). Mild hepatic fibrosis in cholesterol and sodium cholate diet-fed rats. J Vet Med Sci.

[CR21] Kanuri G, Bergheim I (2013). In vitro and in vivo models of non-alcoholic fatty liver disease (NAFLD). Int J Mol Sci.

[CR22] LaBrecque DR, Abbas Z, Anania F, Ferenci P, Khan AG, Goh KL, Hamid SS, Isakov V, Lizarzabal M, Penaranda MM (2014). World gastroenterology organisation global guidelines: nonalcoholic fatty liver disease and nonalcoholic steatohepatitis. J Clin Gastroenterol.

[CR23] Lazo M, Hernaez R, Eberhardt MS, Bonekamp S, Kamel I, Guallar E, Koteish A, Brancati FL, Clark JM (2013). Prevalence of nonalcoholic fatty liver disease in the United States: the Third National Health and Nutrition Examination Survey, 1988–1994. Am J Epidemiol.

[CR24] Lee JY, Kim KM, Lee SG, Yu E, Lim YS, Lee HC, Chung YH, Lee YS, Suh DJ (2007). Prevalence and risk factors of non-alcoholic fatty liver disease in potential living liver donors in Korea: a review of 589 consecutive liver biopsies in a single center. J Hepatol.

[CR25] Lindback SM, Gabbert C, Johnson BL, Smorodinsky E, Sirlin CB, Garcia N, Pardee PE, Kistler KD, Schwimmer JB (2010). Pediatric nonalcoholic fatty liver disease: a comprehensive review. Adv Pediatr.

[CR26] Liu Q, Bengmark S, Qu S (2010). The role of hepatic fat accumulation in pathogenesis of non-alcoholic fatty liver disease (NAFLD). Lipids Health Dis.

[CR27] Loomba R, Sanyal AJ (2013). The global NAFLD epidemic. Nat Rev Gastroenterol Hepatol.

[CR28] Lopez-Velazquez JA, Silva-Vidal KV, Ponciano-Rodriguez G, Chavez-Tapia NC, Arrese M, Uribe M, Mendez-Sanchez N (2014). The prevalence of nonalcoholic fatty liver disease in the Americas. Ann Hepatol.

[CR29] Machado MV, Cortez-Pinto H (2014). Management of fatty liver disease with the metabolic syndrome. Expert Rev Gastroenterol Hepatol.

[CR30] Marchesini G, Bugianesi E, Forlani G, Cerrelli F, Lenzi M, Manini R, Natale S, Vanni E, Villanova N, Melchionda N (2003). Nonalcoholic fatty liver, steatohepatitis, and the metabolic syndrome. Hepatology.

[CR31] Nagarajan P, Mahesh KMJ, Venkatesan R, Majundar SS, Juyal RC (2012). Genetically modified mouse models for the study of nonalcoholic fatty liver disease. World J Gastroenterol.

[CR32] Neuschwander-Tetri BA (2005). Nonalcoholic steatohepatitis and the metabolic syndrome. Am J Med Sci.

[CR33] Qi X, Jia J, Ren W, Yang M, De Stefano V, Wang J, Fan D (2014). Scientific publications on portal vein thrombosis and Budd-Chiari syndrome: a global survey of the literature. J Gastrointestin Liver Dis.

[CR34] Ramos JM, Padilla S, Masia M, Gutierrez F (2008). A bibliometric analysis of tuberculosis research indexed in PubMed, 1997–2006. Int J Tuberc Lung Dis.

[CR35] Rector RS, Thyfault JP, Wei Y, Ibdah JA (2008). Non-alcoholic fatty liver disease and the metabolic syndrome: an update. World J Gastroenterol.

[CR36] Sanyal AJ, Chalasani N, Kowdley KV, McCullough A, Diehl AM, Bass NM, Neuschwander-Tetri BA, Lavine JE, Tonascia J, Unalp A (2010). Pioglitazone, vitamin E, or placebo for nonalcoholic steatohepatitis. N Engl J Med.

[CR37] Shi KQ, Fan YC, Liu WY, Li LF, Chen YP, Zheng MH (2012). Traditional Chinese medicines benefit to nonalcoholic fatty liver disease: a systematic review and meta-analysis. Mol Biol Rep.

[CR38] Sorensen AA (2009). Alzheimer’s disease research: scientific productivity and impact of the top 100 investigators in the field. J Alzheimers Dis.

[CR39] Spruss A, Kanuri G, Wagnerberger S, Haub S, Bischoff SC, Bergheim I (2009). Toll-like receptor 4 is involved in the development of fructose-induced hepatic steatosis in mice. Hepatology.

[CR40] Takahashi Y, Soejima Y, Fukusato T (2012). Animal models of nonalcoholic fatty liver disease/nonalcoholic steatohepatitis. World J Gastroenterol.

[CR41] Tipoe GL, Ho CT, Liong EC, Leung TM, Lau TY, Fung ML, Nanji AA (2009). Voluntary oral feeding of rats not requiring a very high fat diet is a clinically relevant animal model of non-alcoholic fatty liver disease (NAFLD). Histol Histopathol.

[CR42] Ugolini D, Puntoni R, Perera FP, Schulte PA, Bonassi S (2007). A bibliometric analysis of scientific production in cancer molecular epidemiology. Carcinogenesis.

[CR43] van der Poorten D, Milner KL, Hui J, Hodge A, Trenell MI, Kench JG, London R, Peduto T, Chisholm DJ, George J (2008). Visceral fat: a key mediator of steatohepatitis in metabolic liver disease. Hepatology.

[CR44] Vernon G, Baranova A, Younossi ZM (2011). Systematic review: the epidemiology and natural history of non-alcoholic fatty liver disease and non-alcoholic steatohepatitis in adults. Aliment Pharmacol Ther.

[CR45] Vuppalanchi R, Chalasani N (2009). Nonalcoholic fatty liver disease and nonalcoholic steatohepatitis: selected practical issues in their evaluation and management. Hepatology.

[CR46] Williams CD, Stengel J, Asike MI, Torres DM, Shaw J, Contreras M, Landt CL, Harrison SA (2011). Prevalence of nonalcoholic fatty liver disease and nonalcoholic steatohepatitis among a largely middle-aged population utilizing ultrasound and liver biopsy: a prospective study. Gastroenterology.

[CR47] Wu JW, Wang SP, Alvarez F, Casavant S, Gauthier N, Abed L, Soni KG, Yang G, Mitchell GA (2011). Deficiency of liver adipose triglyceride lipase in mice causes progressive hepatic steatosis. Hepatology.

[CR48] Xiao J, Fai SK, Liong EC, Tipoe GL (2013). Recent advances in the herbal treatment of non-alcoholic Fatty liver disease. J Tradit Complement Med.

